# NLRX1 Modulates Immunometabolic Mechanisms Controlling the Host–Gut Microbiota Interactions during Inflammatory Bowel Disease

**DOI:** 10.3389/fimmu.2018.00363

**Published:** 2018-02-26

**Authors:** Andrew Leber, Raquel Hontecillas, Nuria Tubau-Juni, Victoria Zoccoli-Rodriguez, Vida Abedi, Josep Bassaganya-Riera

**Affiliations:** ^1^Landos Biopharma, Inc., Blacksburg, VA, United States; ^2^Nutritional Immunology and Molecular Medicine Laboratory, Biocomplexity Institute of Virginia Tech, Blacksburg, VA, United States; ^3^Department of Biomedical and Translational Informatics, Geisinger Health System, Danville, PA, United States

**Keywords:** inflammatory bowel disease, mucosal immunology, NLRX1, gut microbiome, immunometabolism, intestinal epithelial cells

## Abstract

Interactions among the gut microbiome, dysregulated immune responses, and genetic factors contribute to the pathogenesis of inflammatory bowel disease (IBD). *Nlrx1*^−/−^ mice have exacerbated disease severity, colonic lesions, and increased inflammatory markers. Global transcriptomic analyses demonstrate enhanced mucosal antimicrobial defense response, chemokine and cytokine expression, and epithelial cell metabolism in colitic *Nlrx1*^−/−^ mice compared to wild-type (WT) mice. Cell-specificity studies using cre-lox mice demonstrate that the loss of NLRX1 in intestinal epithelial cells (IEC) recapitulate the increased sensitivity to DSS colitis observed in whole body *Nlrx1*^−/−^ mice. Further, organoid cultures of *Nlrx1*^−/−^ and WT epithelial cells confirm the altered patterns of proliferation, amino acid metabolism, and tight junction expression. These differences in IEC behavior can impact the composition of the microbiome. Microbiome analyses demonstrate that colitogenic bacterial taxa such as *Veillonella* and *Clostridiales* are increased in abundance in *Nlrx1*^−/−^ mice and in WT mice co-housed with *Nlrx1*^−/−^ mice. The transfer of an *Nlrx1*^−/−^-associated gut microbiome through co-housing worsens disease in WT mice confirming the contributions of the microbiome to the *Nlrx1*^−/−^ phenotype. To validate NLRX1 effects on IEC metabolism mediate gut–microbiome interactions, restoration of WT glutamine metabolic profiles through either exogenous glutamine supplementation or administration of 6-diazo-5-oxo-l-norleucine abrogates differences in inflammation, microbiome, and overall disease severity in *Nlrx1*^−/−^ mice. The influence NLRX1 deficiency on SIRT1-mediated effects is identified to be an upstream controller of the *Nlrx1*^−/−^ phenotype in intestinal epithelial cell function and metabolism. The altered IEC function and metabolisms leads to changes in barrier permeability and microbiome interactions, in turn, promoting greater translocation and inflammation and resulting in an increased disease severity. In conclusion, NLRX1 is an immunoregulatory molecule and a candidate modulator of the interplay between mucosal inflammation, metabolism, and the gut microbiome during IBD.

## Introduction

Nod-like receptors (NLRs) play a central role in immune surveillance at the gut mucosa by facilitating detection, recognition, and discrimination of metabolic and microbial components at the gut mucosa (1). NLR dysregulation has been associated with inflammatory bowel disease (IBD) (2). Genetic polymorphisms in several NLRs have been described in IBD such as NLRC1 (NOD1) and NLRC2 (NOD2) (3). Negative-regulatory NLRs, NLRC3, NLRP12 and nucleotide-binding oligomerization domain, leucine-rich repeat containing X1 (NLRX1) (4) exert anti-inflammatory activities mediated in part through NF-κB (5–7) antagonism.

NLRX1 is a mitochondria-associated NLR linked to the regulation of inflammation, autophagy, and reactive oxygen species production in response to viral and bacterial pathogens, such as downregulating inflammation following viral infection (8) and regulating interferon production (9). While NLRX1 is mostly classified as a negative regulatory NLR, contradictory evidence suggests that it may be crucial in the establishment of reactive oxygen species production, generally considered to be a pro-inflammatory response (10). Meanwhile, NLRX1 controls metabolism in addition to the function of CD4+ T cells (11). It remains largely unknown whether the loss of NLRX1 signaling impairs central gut mucosal immunoregulatory mechanisms at the interface of host response, microbiome, and diet that impact sensitivity to IBD. Understanding such potential immunometabolic mechanisms might be critical for validating NLRX1 as a potential new therapeutic target for IBD.

Dysregulated immune responses that impair tolerance between the host, gut microbiota, and environment perpetuate a tissue-damaging chronic inflammatory state. However, the fundamental mechanisms of interaction among gut microbiota, environment, and genes have not been elucidated (12). NLRX1 holds promise as a gut mucosal sensor of inflammation, microbial changes, and metabolic disruption implicated in regulating the flux of information between the gut microbiota, dietary factors, and the immune response. The metabolism of glutamine is needed for both the inflammatory actions of macrophages (13) and the differentiation of effector T cells (14). In addition, proliferating cells in epithelial crypts upregulate the usage of glutamine and decreases upon maturation to the villi (15). To suppress inflammation, IBD patients rely on life-long modestly successful treatment plans, many of which are accompanied by undesirable side effects of biologics, including cancer, infection, and death (16). Thus, identifying novel potential immunoregulatory targets such as NLRX1 that modulate tolerance and immune-mediated intestinal inflammation whose expression can be pharmacologically manipulated is significant for addressing an unmet medical need for safer, more effective, and faster IBD therapeutics.

The gut microbiome plays an important role in modulating gut pathology and disease outcomes in IBD (17). A decrease in bacterial diversity and in the relative abundance of *Bacteroidetes* taxa has been commonly observed in IBD patients (18). With the decrease in common commensals, an expansion of *Proteobacteria* and a bloom in pathogenic species such as *Escherichia coli* and *Clostridium difficile* are apparent in patients experiencing active disease (19, 20). Alterations in the gut microbiome can also change the bacterial metabolic profiles in the gut (21, 22), thereby modulating the composition of nutrients and small molecules, such as lipids or amino acids, locally available to intestinal epithelial cells (IEC) (23) with dietary and endogenously generated metabolites implicated in the prevention and resolution of inflammation (24). We have previously determined that some dietary lipids such as conjugated trienes, and n-3 PUFA are low-affinity ligands of NLRX1 (25). However, host microbial sensors that can modulate the sequestration and secretion of metabolic substrate are currently unknown.

This study provides novel initial evidence *in vivo* that NLRX1 may serve as a central regulatory hub of gut homeostasis by modulating mucosal immunity, metabolism, and gut microbiome composition. Cell-specificity studies using cre-lox conditional knockout mice lacking NLRX1 in IEC suggest that the dysregulation of NLRX1 in the gut mucosa and on IEC can predispose to a reactive and pro-inflammatory state at the colonic mucosa. Active communication between the colonic epithelium, the lamina propria leukocytes, and the gut microbiota through NLRX1 results in altered metabolic profiles and antimicrobial peptide production. NLRX1 controls NAD+ cycling and SIRT1 signaling to maintain epithelial barrier function and prevent self-perpetuating cycles increased intestinal permeability and exacerbated inflammatory responses. This article examines NLRX1-mediated host immunometabolic mechanisms of microbial control, the resulting shift in gut microbiome constituents in *Nlrx1^−/−^* mice, and the implications of these shifts in disease and pathology using mouse models of epithelial cell injury.

## Results

### NLRX1-Deficiency Is Associated with Enhanced Colonic Inflammatory Chemokine Production and Antimicrobial Peptide Expression

**Figure 1:**
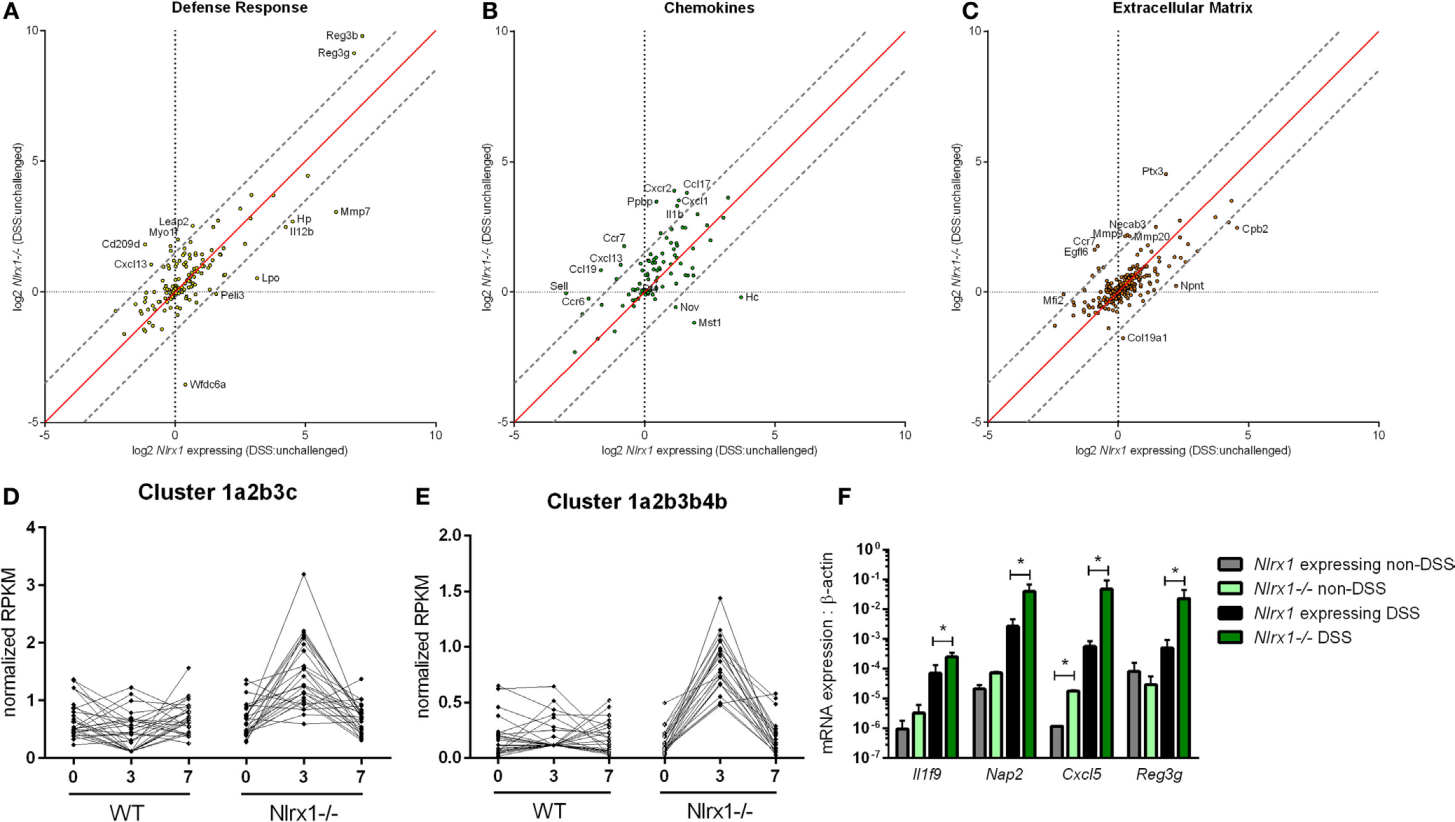
RNA-seq analysis of colons from unchallenged and DSS-challenged *Nlrx1* expressing and *Nlrx1^−/−^* mice indicates differences in gene expression. The log2 changes between DSS-challenged and unchallenged samples were calculated and plotted for each genotype within functional families of genes **(A–C)**. Each point within the graph represents an individual gene. Points falling near the red identity line in each plot correspond to those that have similar response patterns in *Nlrx1*-expressing and *Nlrx1^−/−^* animals. The gray dotted lines follow a 1.5 log2 difference from the identity line. Genes outside of the gray lines were considered to have different behavior between genotypes. Clusters enriched in proliferation **(D)** and metabolic **(E)** functions following hierarchical clustering for temporal patterns within RNA-seq dataset. Quantitative real-time PCR validation of cytokine, chemokine, and antimicrobial peptide expression normalized to beta-actin **(F)**. Asterisks (*) mark significance (*p* ≤ 0.05) in comparison between treatments of the same genotype (*n* = 8), experiment in triplicate. Number signs (^#^) mark significance (*p* ≤ 0.05) between *Nlrx1^−/−^* and *Nlrx1*-expressing only (*n* = 8), experiment in triplicate.

To gain a global systems-wide understanding on molecular associations between NLRX1 and gut inflammation, we performed RNA sequencing on colons from *Nlrx1*-expressing and *Nlrx1^−/−^* mice at days 3 and 7 of DSS challenge. In accordance with histopathological findings (Figure S1 in Supplementary Material), *Nlrx1^−/−^* mice had enriched pro-inflammatory responses (*Il6, Il11, Il1b)* correlating to elevated NF-κB signaling (Figure S2 in Supplementary Material). Notably, *Nlrx1^−/−^* mice exhibited dysregulation in functions associated with the recruitment of immune cells and interactions with the microbiome (Figures 1A–C). While many of the inflammatory chemokines with observable genotype effects were slightly upregulated even in *Nlrx1^−/−^* mice without administration of DSS, the differences were more accentuated following DSS treatment. Specifically, neutrophil-attracting chemokines, *Nap2* and *Cxcl5*, were upregulated without DSS treatment and further increased on day 7 of DSS challenge, verified by qRT-PCR (Figure 1F). In contrast, the expression of antimicrobial peptides, highlighted by *Reg3g*, was slightly downregulated in the colons of *Nlrx1^−/−^* non-DSS mice compared to their *Nlrx1*-expressing counterparts prior to a large upregulation in mRNA expression on day 7 of DSS challenge (Figure 1F). Additionally, a subset of these defense response genes, including *Csf3, Ctsg*, and *Il17c*, were detected in observable quantities only in colitic *Nlrx1^−/−^* colonic samples. Hierarchical clustering of the RNA-seq dataset was used to identify differences in temporal expression patterns (Figure S3 in Supplementary Material). After hierarchical clustering, 3 of the 15 clusters were enriched in cell life cycle-associated functions. A single cluster (Figure 1D), defined by an upregulation of expression on day 3 in *Nlrx1^−/−^* identified an increased prevalence of proliferation and cell division through growth factors (*Igf2bp2, Pdgfb*), transcription factors promoting NF-κB and Wnt signaling (*Carm1, Paf1*) and factors responsible for progressing the cell cycle (*Klhl22, Skp2, Ska3*). Further, a cluster with similar expression patterns (Figure 1E) was identified to be associated with metabolic functions including genes associated with amino acid (*Glud1, Got1, Gpt*) and phosphate (*Mmd2, Galr1, Oprk1*) metabolism.

### Increased Sensitivity to DSS Colitis Is a Result of Loss of NLRX1 in Epithelial Cells

**Figure 2:**
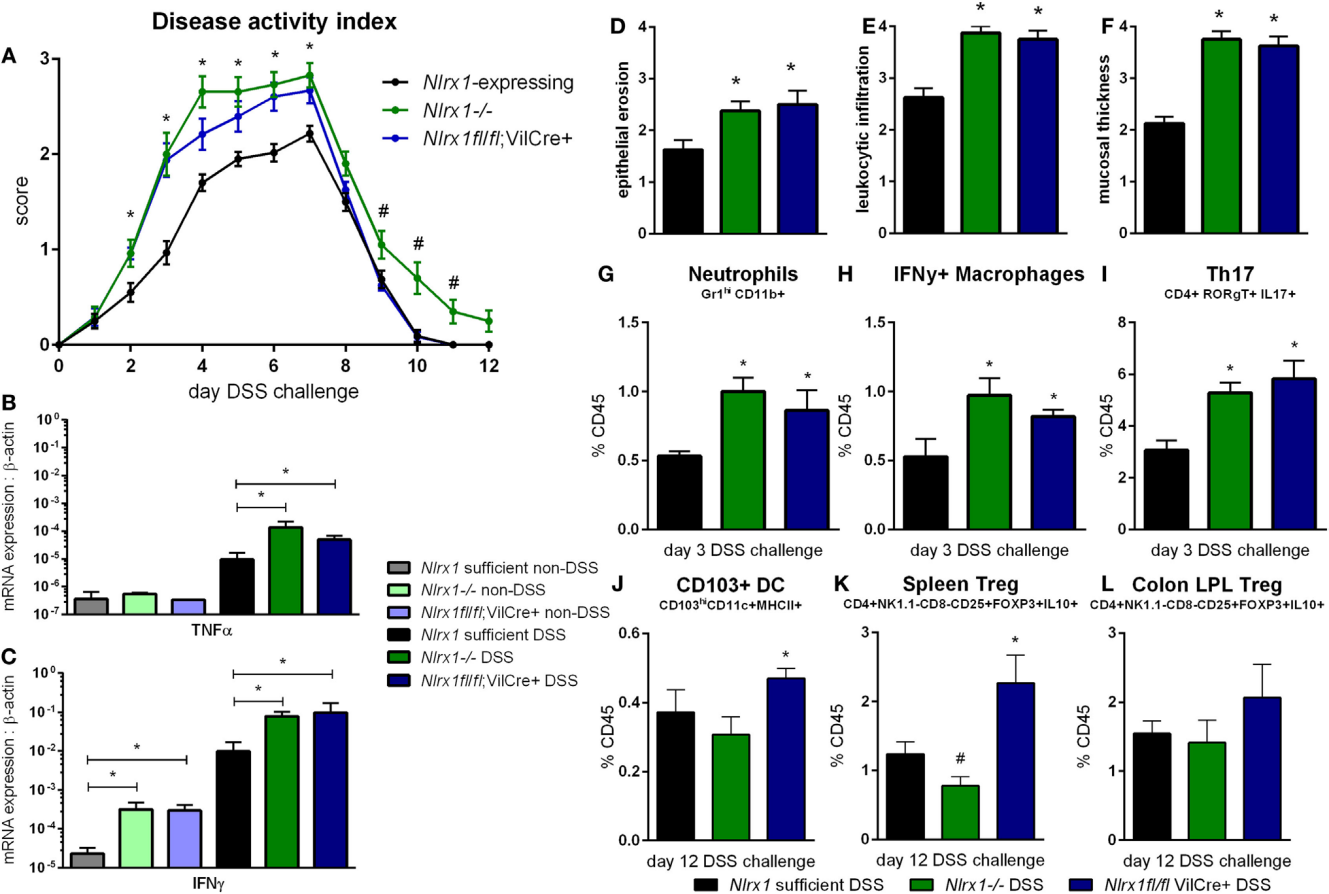
Epithelial cell NLRX1 knockout mice recapitulate the phenotype of whole body NLRX1*^−/−^*. *Nlrx1*-expressing, *Nlrx1^−/−^* and *Nlrx1fl*/*fl*; VillinCre+ mice were challenged with DSS. Increases in disease activity index were observed both with *Nlrx1^−/−^* and *Nlrx1fl*/*fl*; VillinCre+ mice compared to *Nlrx1*-expressing **(A)**. *Nlrx1^−/−^* and *Nlrx1fl*/*fl*; VillinCre+ animals possess significantly higher mRNA expression of TNFα **(B)** and IFNγ **(C)** at day 7 of DSS challenge compared to *Nlrx1*-expressing. Sections of colonic tissue were obtained and histologically graded on a scale of 0–4 at day 7 of DSS challenge. Significant increases in epithelial erosion **(D)**, leukocytic infiltration **(E)**, and mucosal thickness **(F)** were observed in *Nlrx1^−/−^* and *Nlrx1fl*/*fl*; VillinCre+ mice compared to *Nlrx1*-expressing colonic neutrophils **(G)**, IFNγ-producing macrophages **(H)**, and Th17 cells **(I)**, as percentage of CD45+ cells, on day 3 of DSS challenge. Flow cytometry analysis of colonic CD103+ DC **(J)**, splenic Treg **(K)** and colonic Treg **(L)** as percentages of CD45+ cells on day 12 of DSS challenge Asterisks (*) mark significance (*p* ≤ 0.05) in comparison between genotypes of the same treatment (*n* = 8). Number signs (^#^) mark significance (*p* ≤ 0.05) between *Nlrx1^−/−^* and *Nlrx1*-expressing only (*n* = 8).

Based on the gene expression patterns within the colon, the experimental model of colitis was tested on VillinCre-lox mice (26). The increased severity of disease previously observed in *Nlrx1^−/−^* mice, compared to *Nlrx1*-expressing mice, was also observed within the *Nlrx1fl*/*fl*; VillinCre+ group through the time period of DSS administration compared to *Nlrx1*-expressing littermate controls (Figure 2A). The greatest differences in disease activity indices (DAIs) occurred on day 3 of DSS challenge suggesting that epithelial NLRX1 might be particularly critical early in the response to challenge, but continued through the active DSS period. However, unlike *Nlrx1^−/−^, Nlrxfl/fl;* VillinCre+ mice quickly recovered post-DSS on days 8 through 12, with little to no differences observable between *Nlrx1fl/fl*; VillinCre+, and *Nlrx1*-expressing littermates, a previously unreported finding. On day 7 of DSS challenge, the significantly increased expression of inflammatory cytokines, TNFα and IFNγ, in *Nlrx1^−/−^* mice, compared to *Nlrx1*-expressing was also observed within the *Nlrx1fl*/*fl*; VillinCre+ group (Figures 2B,C). Further, the observable histological differences in mucosal thickness, epithelial erosion, and leukocytic infiltration within the *Nlrx1fl*/*fl*; VillinCre+ group were consistent with the *Nlrx1^−/−^* colons (Figures 2D–F), suggesting that epithelial cell NLRX1 accounts for the majority of phenotypic differences observed in the whole body *Nlrx1^−/−^* mice during active DSS challenge, days 0 through 7. Further evidence from immunophenotyping of colonic lamina propria lymphocytes at day 3 also supports this claim. Similar to Nlrx1*^−/−^* mice, *Nlrx1fl*/*fl*; VillinCre+ mice displayed greater percentages of neutrophils (Figure 2G), CD4+ Th17 cells (Figure 2H), and IFNγ-producing macrophages (Figure 2I) at day 3 of DSS challenge compared to *Nlrx1*-expressing littermates. In contrast, *Nlrx1fl*/*fl*; VillinCre+ mice have increased CD103+ dendritic cells (Figures 2J–L) at day 12 of DSS challenge. While counterintuitive to the *Nlrx1^−/−^* phenotype, the greater proportion of regulatory and homeostatic cell types at this phase in the experimental timeline is in line with the observed trend of accelerated recovery in *Nlrx1fl*/*fl*; VillinCre+ mice (Figure 2A). Potentially, faster epithelial cell proliferation in *Nlrx1fl*/*fl*; VillinCre+ mice may lead to increased healing of lesions in the absence of *Nlrx1^−/−^* immune cells that prolong inflammation after removal of the causative agent (DSS) on day 7. This suggests that NLRX1 deficiency in IEC can trigger and recapitulate events during active inflammation, but cooperation between IEC and immune cells creates a cumulative effect in initiation, propagation, and recovery processes in IBD.

### Altered Antimicrobial Responses Correlate to Shifts in Microbial Communities

**Figure 3:**
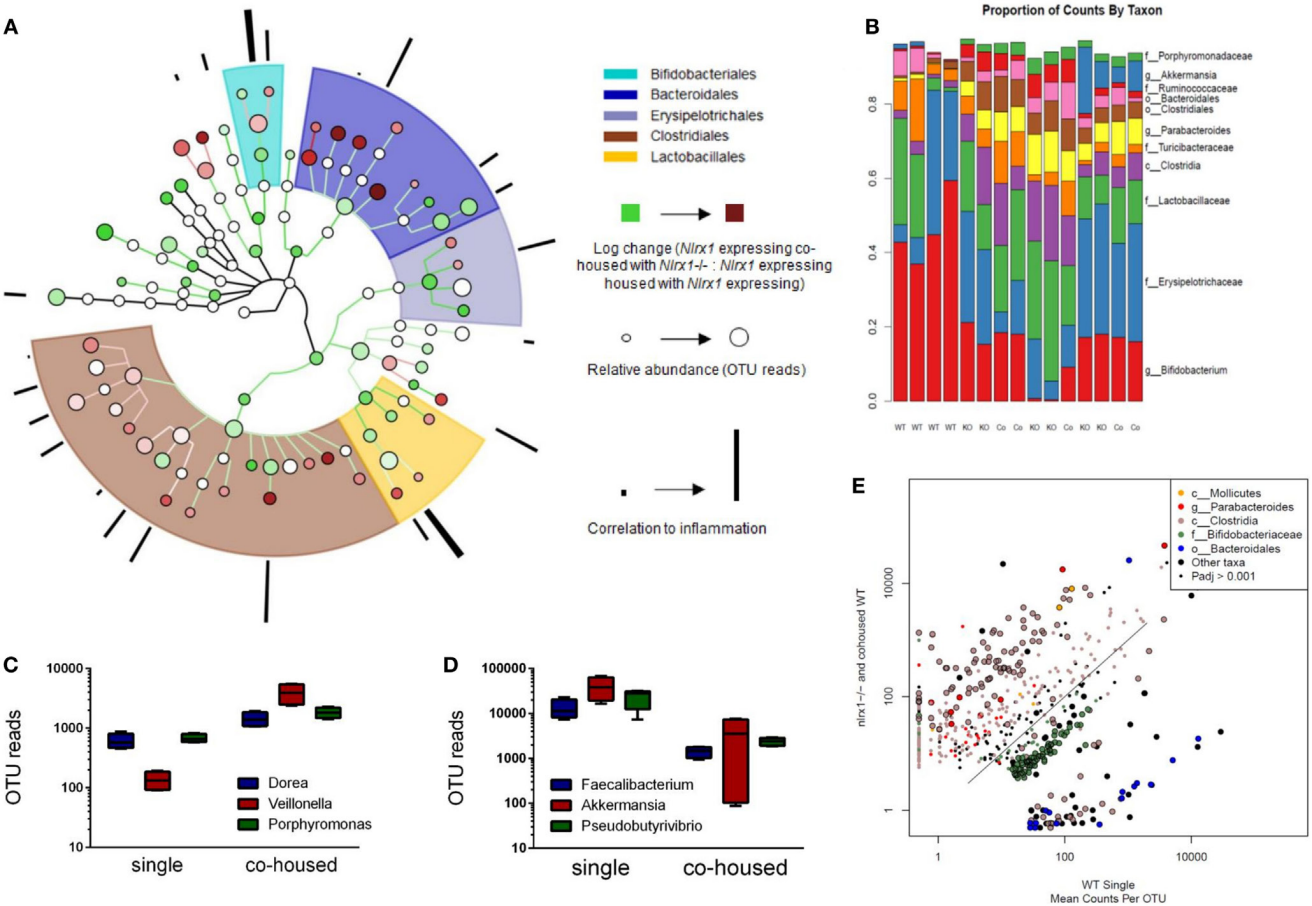
The loss of NLRX1 results in transferrable changes in the gut microbiota composition. *Nlrx1*-expressing mice were housed only with wild-type mice (single) or co-housed with *Nlrx1^−/−^* mice (co-housed) (*n* = 4). After this period, colonic contents were collected for 16S rRNA-based sequencing. Observed taxa were compiled into a phylogenetic map using the Python-based program, Graphlan **(A)**. Microbiota composition was comprised largely of five main order *Clostridiales*, Bacteroidales, *Lactobacillales, Erysipelotrichales*, and *Bifidobacteria* indicated by the shaded ring sections. The relative operational taxonomic units (OTUs) in co-housed compared to single are indicated by the interior color of each taxon marker on a scale from green (taxon lower in co-housed) to red (taxon higher in co-housed). The size of each marker is dependent on the number of reads observed. On the outer ring, the correlation to inflammation based on a search algorithm for genera and species are marked in a bar graph style. A stacked bar graph for individual samples displays changes between single and co-housed samples and the high degree of agreement between co-housed *Nlrx1*-expressing and their *Nlrx1^−/−^* counterparts **(B)**. Boxplots of increased **(C)** and decreased **(D)** taxa following co-housing. Scatterplot of main taxa displaying altered abundance of Clostridia and Bacteroidales orders between *Nlrx1^−/−^* and *Nlrx1*-expressing samples **(E)**.

Due to differences in antimicrobial peptide responses and epithelial cell behavior observed in global transcriptomics analyses, we sought to determine the interplay between NLRX1, immunity, and the colonic microbiota. Genetic deletion of PRRs is linked to alterations in microbial ecology that drive enhanced susceptibility and exacerbated immunopathology during autoimmune diseases. 16S rRNA-based sequencing was performed on colonic contents obtained from *Nlrx1^−/−^* and *Nlrx1*-expressing littermate mice at 8 weeks of age. *Nlrx1*-expressing mice were either housed only with other *Nlrx1*-expressing mice or co-housed with *Nlrx1^−/−^* mice for 5 weeks from date of wean prior to sample collection. Following this period, a noticeable shift in bacterial composition is observable (Figure 3). The majority of the microbial population was comprised of bacteria sourced from the *Bacteroidetes* and *Firmicutes* phyla. Many of the predominant populations differentially abundant in the *Nlrx1*-expressing co-housed with *Nlrx1^−/−^* group compared to *Nlrx1*-expressing controls were observed within the *Clostridiales* order (Figures 3A,E). A comparison of changes following co-housing to the microbiome of independent *Nlrx1*^+/+^ and *Nlrx1^−/−^* groups indicates that the gut microbiome following co-housing closely resembles that of *Nlrx1^−/−^* mice (Figure 3B) with notable decreases in *Bifidobacterium* and Bacteroidales taxa and increases in *Ruminococcaceae* and *Porphyromonadaceae*. Bacterial taxons most highly associated with inflammation were largely comprised of the groups elevated within *Nlrx1*-expressing mice co-housed with *Nlrx1^−/−^*. This was displayed by the greater association of perimeter bars with the red taxa (co-housed increased) as opposed to green taxa (co-housed decreased), which was used as an estimate of the overall profile rather than insight on each particular taxa (Figure 3A). More specifically, large increases were seen within *Dorea, Veillonella*, and *Porphyromonas* groups (Figure 3C). Taxa with observable relative decreases after co-housing with *Nlrx1^−/−^* mice were *Faecalibacterium, Akkermansia*, and *Pseudobutyrivibrio* (Figure 3D).

### Transfer of *Nlrx1^−/−^* Gut Microbiome to *NLRX1+/+* Mice Worsens Disease Activity in IBD

**Figure 4:**
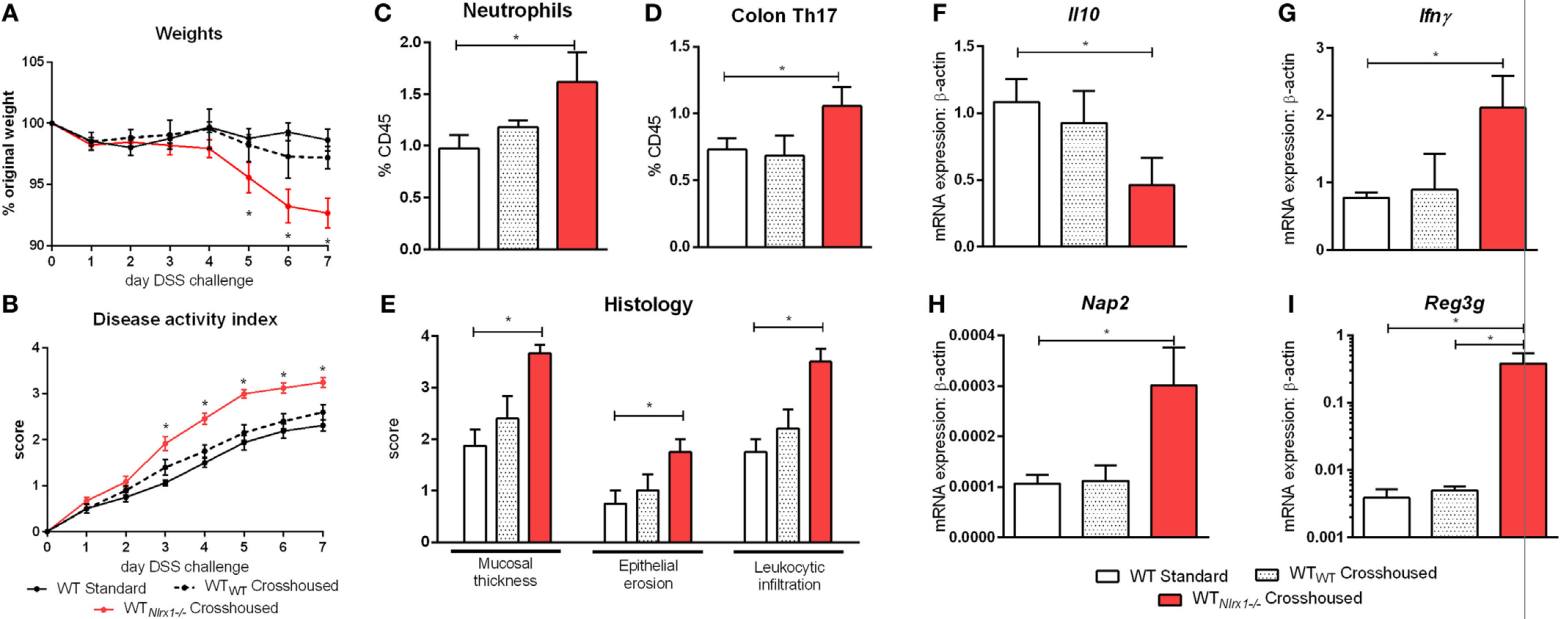
Nlrx1*^−/−^* microbiome transfer worsens colitis in NLRX1-expressing mice. Wild-type mice were given antibiotics then co-housed with non-antibiotic administered mice. Weight change **(A)** and disease activity index **(B)** of non-antibiotic administered mice wild-type mice (WT Standard), antibiotic administered WT mice housed with non-antibiotic WT mice (WT_WT_), and antibiotic administered WT mice housed with non-antibiotic *Nlrx1^−/−^* mice (*WT_Nlrx1_^−/−^*) following cohousing. Colonic neutrophil **(C)** and Th17 **(D)** responses on day 7 of DSS challenge. Colonic histological scores **(E)** for mucosal thickness, epithelial erosion, and leukocytic infiltration criteria on day 7 of DSS challenge. Expression of Il10 **(F)**, Ifnγ **(G)**, Nap2 **(H)**, and Reg3g **(I)** mRNA by qRT-PCR assay of colon samples on day 7 of DSS challenge. Asterisks (*) mark significance (*p* ≤ 0.05) in comparison between treatments within genotypes (*n* = 6).

Due to currently insufficient characterization of host–microbiome interactions and multitude of contributing effects, the characterization of one microbiome profile or a single species as pro-inflammatory or anti-inflammatory can only provide a rough estimate of the microbial phenotype. Therefore, we aimed to classify the effects of the *Nlrx1^−/−^* microbiome on disease response by microbiome transfer. Wild type and *Nlrx1^−/−^* mice were raised separately following weaning. Prior to DSS challenge, WT (*Nlrx1*^+/+^) and *Nlrx1^−/−^* groups were administered a gut microbiota-depleting antibiotic cocktail. Antibiotic administered mice were then cross-cohoused with mice of the opposite genotype that had not received antibiotics. The gut microbiome was repopulated for a 2-week period prior to initiating the DSS challenge. Wild-type mice given antibiotics and housed with *Nlrx1^−/−^* mice (*WT_Nlrx1_^−/−^*) experienced significantly greater weight loss and disease activity scores than standard WT housed in single genotype cages and WT mice given antibiotics and housed with WT mice (WT_WT_) (Figures 4A,B). Colons of *WT_Nlrx1_^−/−^* displayed marked increases in mucosal thickness, epithelial erosion, and leukocytic infiltration upon histological evaluation of colonic specimens when compared to those of conventional WT mice and WT_WT_ (Figure 4E). Further, increases in inflammatory cell types, neutrophils (Gr1+CD11b+), and Th17 (CD4+IL17+RORγT+) were observed in WT_Nlrx1_*^−/−^* compared to conventional WT and WT_WT_ (Figures 4C,D). At the molecular level, cross-cohousing significantly decreased *Il10* expression while increasing *Ifn*γ, *Nap3*, and *Reg3g* expression within *WT_Nlrx1_^−/−^* (Figures 4F–I). The reverse transfer in which *Nlrx1^−/−^* were treated with antibiotics and co-housed either with WT or *Nlrx1^−/−^* mice was conducted (Figure S4 in Supplementary Material). A partial amelioration of increased disease severity was observed in *Nlrx1^−/−^*_WT_ suggesting that either the WT microbiome is only transiently stable in *Nlrx1^−/−^* or additional contributing factors inherent in the *Nlrx1^−/−^* dysregulated host immune responses also contribute to disease severity.

### NLRX1-Deficient Epithelial Cells Display Altered Metabolism, Proliferation, and Expression of Tight Junctions

**Figure 5:**
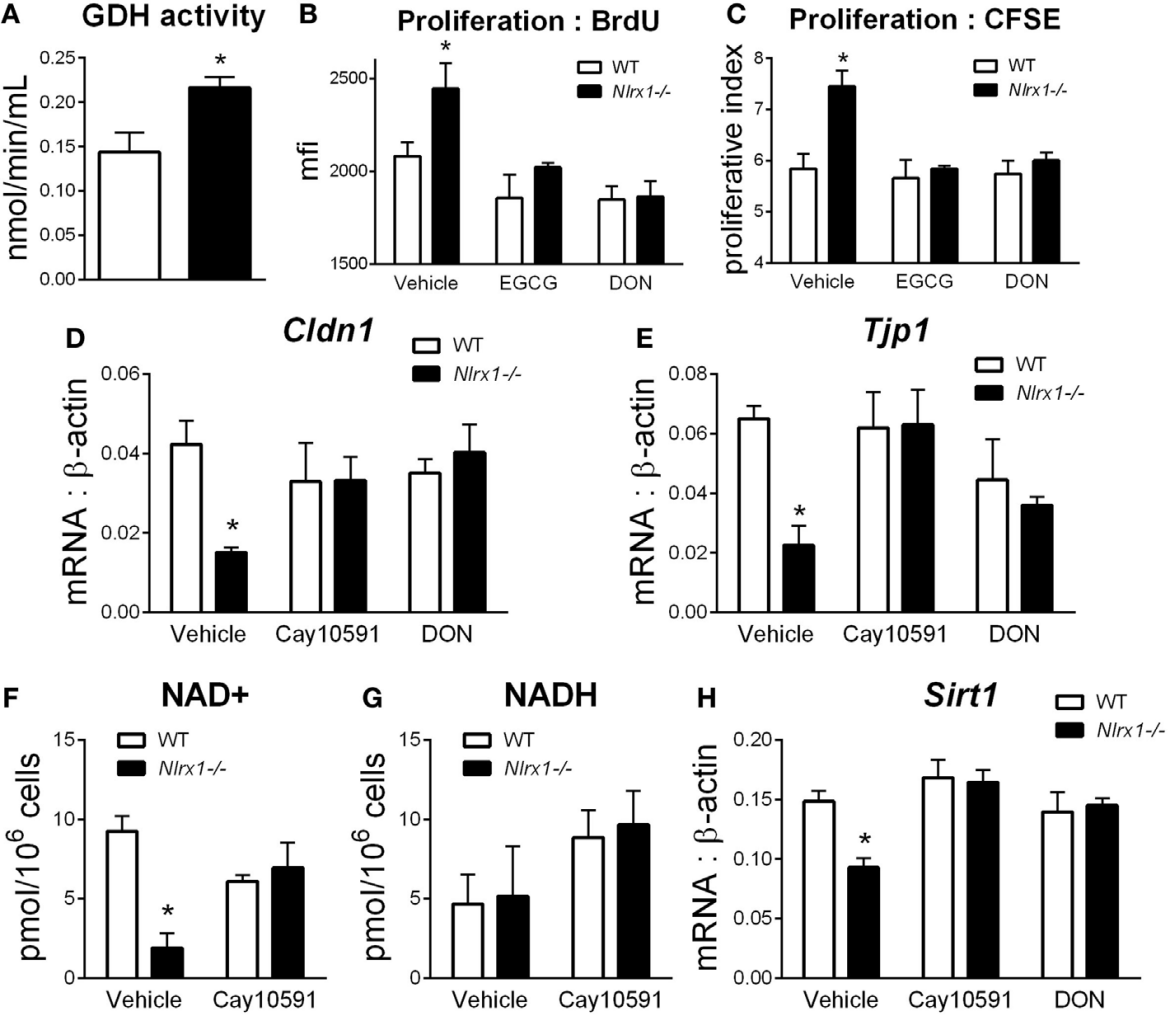
Characterization of NLRX1 deficiency in the metabolic and expression profiles of intestinal organoids. Glutamate dehydrogenase activity by colorimetric assay of organoid homogenate **(A)**. Proliferation of intestinal organoids by BrdU **(B)** and CFSE **(C)** staining. Expression of Cldn1 **(D)** and Tjp1 **(E)** after 24 h of LPS stimulation normalized to beta-actin. Concentration of NAD+ **(F)** and NADH **(G)** by colorimetric assay. Expression of Sirt1 **(H)** after 24 h of LPS stimulation normalized to beta-actin. Asterisks (*) mark significance (*p* ≤ 0.05) in comparison between genotypes. Data are a result of three independent experiments, each containing four replicates.

To identify additional contributing factors of Nlrx1 deficiency on epithelial cell behavior, we assessed whether the proliferation and metabolic changes were present in epithelial cells through an intestinal organoid system, which has proven to show greater translational application to *in vivo* responses than traditional culture of primary cells (27, 28), particularly, within studies of proliferation and metabolism. From RNA-sequencing (Figure 1E), metabolically, differences between Nlrx1*^−/−^* and WT mice existed within amino acid metabolism, specifically glutamine metabolism (*Glud1, Got1, Gpt*). Therefore, the activity of glutamate dehydrogenase was used as a starting point for this analysis. Glutamate dehydrogenase activity was significantly increased in *Nlrx1^−/−^* intestinal organoids (Figure 5A). The proliferation of the intestinal crypts was increased in *Nlrx1^−/−^* samples *via* measurement by BrdU and CFSE staining (Figures 5B,C) with observed decreases in tight junction gene expression (Figures 5D–E). In a combination of the two findings, inhibition of glutamate dehydrogenase by established inhibitors epigallocatechin gallate and DON rescued the augmented proliferation of *Nlrx1^−/−^* intestinal organoids (Figures 5B,C). Linked to the altered glutamine metabolism, *Nlrx1^−/−^* intestinal organoids had lower levels of NAD+ without changes in NADH levels indicating altered cycling or consumption of NAD+ (Figures 5F,G). Similar trends in NAD+ levels were observed *in vivo* within whole colon (Figure S5 in Supplementary Material). Sirtuins are a key signaling pathway dependent on NAD+ levels that are linked to inflammation, metabolism, and epithelial cell behavior. Notably, the expression of two members of the sirtuin family (Sirt1, Sirt3) were significantly lower in the colons of Nlrx1*^−/−^*, while three others showed similar downregulation trends (Figure S6 in Supplementary Material). The expression of Sirt1 was decreased in *Nlrx1^−/−^* intestinal organoids (Figure 5H) suggesting that NLRX1 balancing of NAD+ cycling influences sirtuin-mediated control of epithelial cell metabolism and barrier maintenance. Further, administration of the Sirt1 activator, Cay10591, abrogates differences in NAD+ levels (Figure 5H).

### Oral Administration of l-Glutamine to Mice with DSS Colitis Rescues Microbial and Immunological Phenotypes of *Nlrx1* Deficient Mice

**Figure 6:**
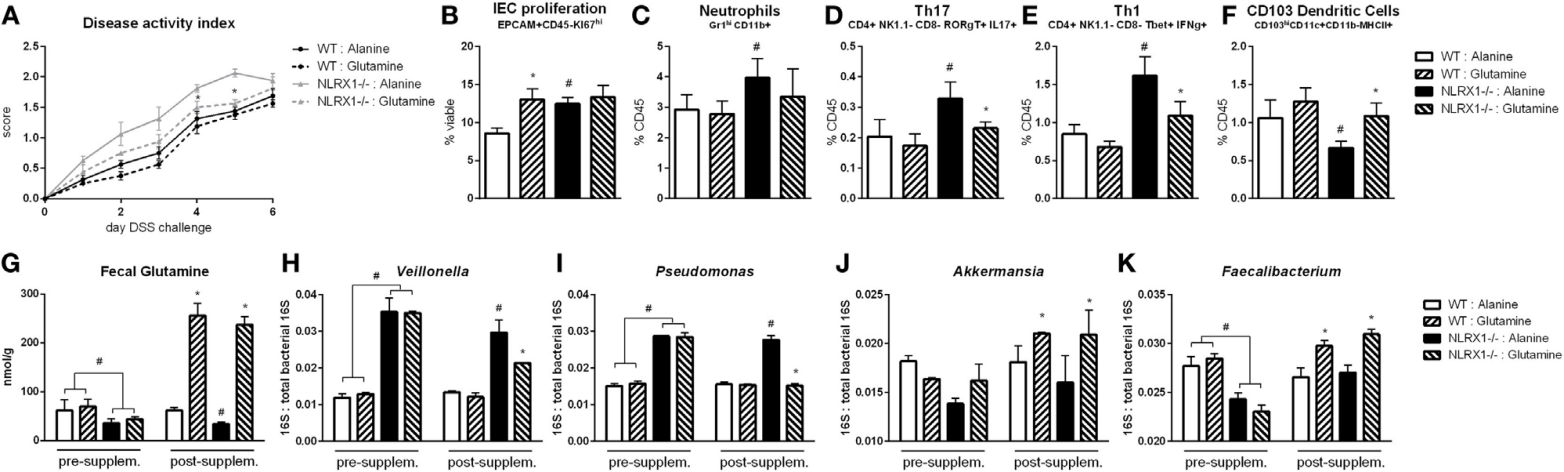
Glutamine supplementation abrogates *Nlrx1^−/−^* associated microbiome. Mice were given daily l-glutamine or alanine by oral gavage prior to DSS. *Nlrx1^−/−^* mice given l-glutamine displayed lower disease activity scores **(A)**. Flow cytometry assessment of intestinal epithelial cell (IEC) proliferation **(B)**, neutrophils **(C)**, Th17 **(D)**, Th1 **(E)**, and CD103+ dendritic cells **(F)** on day 7 of DSS challenge. Fecal glutamine concentration pre- and post-supplementation by colorimetric assay **(G)**. 16S abundance of *Veillonella* **(H)**, *Porphyromonas* **(I)**, *Akkermansia* **(J)**, and *Faecalibacterium* **(K)** by qRT-PCR normalized to total 16S abundance. Asterisks (*) mark significance (*p* ≤ 0.05) in comparison between treatments of the same genotype (*n* = 8). Number signs (^#^) mark significance (*p* ≤ 0.05) between *Nlrx1^−/−^* and *Nlrx1*-expressing only (*n* = 8).

Based on the RNA-seq analysis and subsequent validation, *Nlrx1^−/−^* mice, and specifically *Nlrx1^−/−^* IEC, exhibit an increased utilization of glutamine and other amino acids. With increased host usage, the gut microbiome may have decreased exposure to amino acids leading to expansion of bacteria enriched in amino acid production or non-reliant on environmental amino acids for energy. First, the decreased concentration of fecal glutamine was validated prior to oral administration of glutamine (Figure 6G). WT and *Nlrx1^−/−^* mice were given 375 mg/kg l-glutamine or alanine daily by oral gavage for a 7-day period prior to beginning DSS challenge. Alanine has previously been validated as a negative control in amino acid supplementation studies (29), while a dose titration of glutamine (Figure S7 in Supplementary Material) was used to identify an oral dose sufficient to reduce *Nlrx1^−/−^* v. WT immunological differences without significant effects on WT behavior. Fecal samples were collected before and after this supplementation period for assessment of gut microbial changes. Four taxa (*Veillonella, Porphyromonas, Akkermansia, Faecalibacterium*) identified by 16S sequencing were validated to recapitulate the patterns observed in 16S sequencing pre-supplementation and have reversals of the patterns post-supplementation (Figures 6H–K). Fecal glutamine levels were changed by oral supplementation of glutamine (Figure 6G). During DSS challenge, *Nlrx1^−/−^* mice-administered l-glutamine had lower disease activity than *Nlrx1^−/−^* alanine controls (Figure 6A). While epithelial cell proliferation was increased by glutamine supplementation in WT mice (Figure 6B), cellular markers of inflammation, neutrophils, Th17, and Th1 effector cells were decreased following supplementation (Figures 6C–E). The suppression of CD103+ dendritic cells observed in the *Nlrx1^−/−^* colonic lamina propria was also rescued by l-glutamine supplementation (Figure 6F).

### Inhibition of Host Amino Acid Metabolism Increases Fecal Glutamine to Suppress Gut Microbiome Differences and Inflammation between WT and NLRX1^*−/−*^ Mice

**Figure 7:**
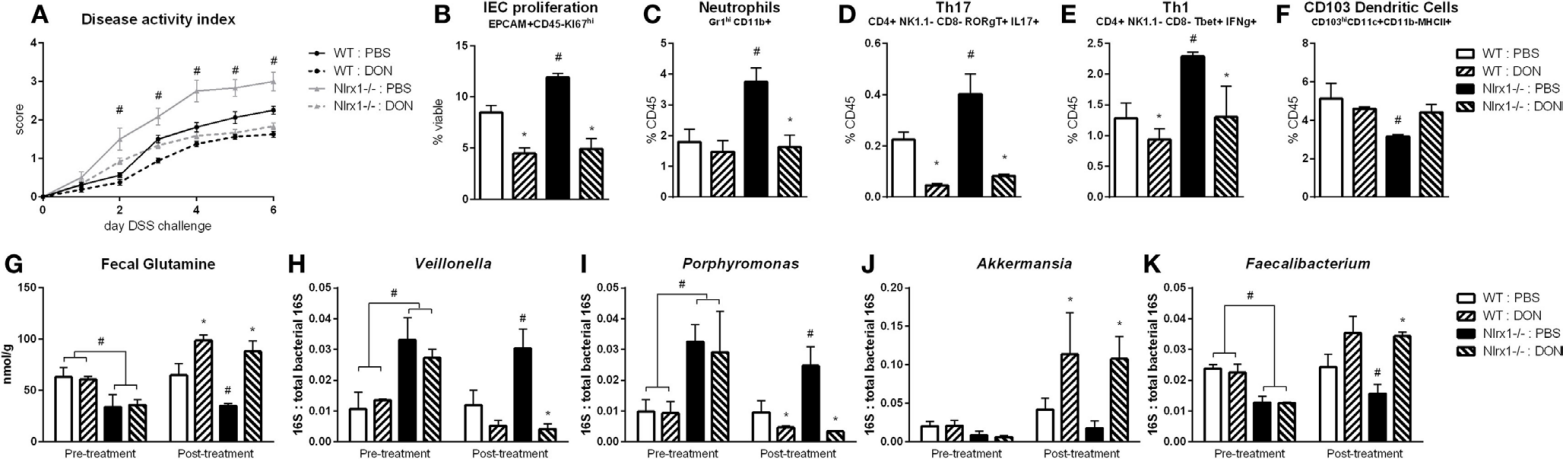
Host amino acid metabolism inhibition abrogates *Nlrx1^−/−^* associated microbiome. Mice were given 6-diazo-5-oxo-l-norleucine (DON) or PBS by intraperitoneal injection every 3 days starting prior to DSS. *Nlrx1^−/−^* mice given DON displayed lower disease activity scores **(A)**. Flow cytometry assessment of intestinal epithelial cell (IEC) proliferation **(B)**, neutrophils **(C)**, Th17 **(D)**, Th1 **(E)**, and CD103+ dendritic cells **(F)** on day 7 of DSS challenge. Fecal glutamine concentration pre- and post-supplementation by colorimetric assay **(G)**. 16S abundance of *Veillonella* **(H)**, *porphyromonas* **(I)**, *Akkermansia* **(J)**, and *Faecalibacterium* **(K)** by qRT-PCR normalized to total 16S abundance. Asterisks (*) mark significance (*p* ≤ 0.05) in comparison between treatments of the same genotype (*n* = 8). Number signs (^#^) mark significance (*p* ≤ 0.05) between *Nlrx1^−/−^* and *Nlrx1*-expressing only (*n* = 8).

With the finding that increased l-glutamine exposure could be beneficial to the gut microbiome, we explored if decreased host utilization of glutamine could provide similar effects. Prior to DSS challenge, WT and *Nlrx1^−/−^* mice were treated with 1.3 mg/kg 6-diazo-5-oxo-l-norleucine (DON) every third day by intraperitoneal injection. DON treatment resulted in increased fecal glutamine in both WT and *Nlrx1^−/−^* with no significant differences existing between WT treated with DON and *Nlrx1^−/−^* treated with DON (Figure 7G). As with the l-glutamine supplementation, DON treatment resulted in decreased *Veillonella* and *Porphyromonas* with increased *Akkermansia* and *Faecalibacterium* (Figures 7H–K). During DSS challenge, DON-treated *Nlrx1^−/−^* groups showed no statistical differences from either WT groups and had significantly less DAI than standard *Nlrx1^−/−^* groups (Figure 7A). With the inhibition of host amino acid metabolism, the proliferation of colonic epithelial cells was significantly decreased in WT and *Nlrx1^−/−^* mice (Figure 7B). Colonic neutrophils, Th17, and Th1 cells were decreased with DON treatment while CD103+ dendritic cells were slightly increased (Figures 7C–F).

### Loss of NLRX1 Contributes to Altered Glutamine Metabolism and Inflammatory Response *via* a SIRT1-Dependent Mechanism

**Figure 8:**
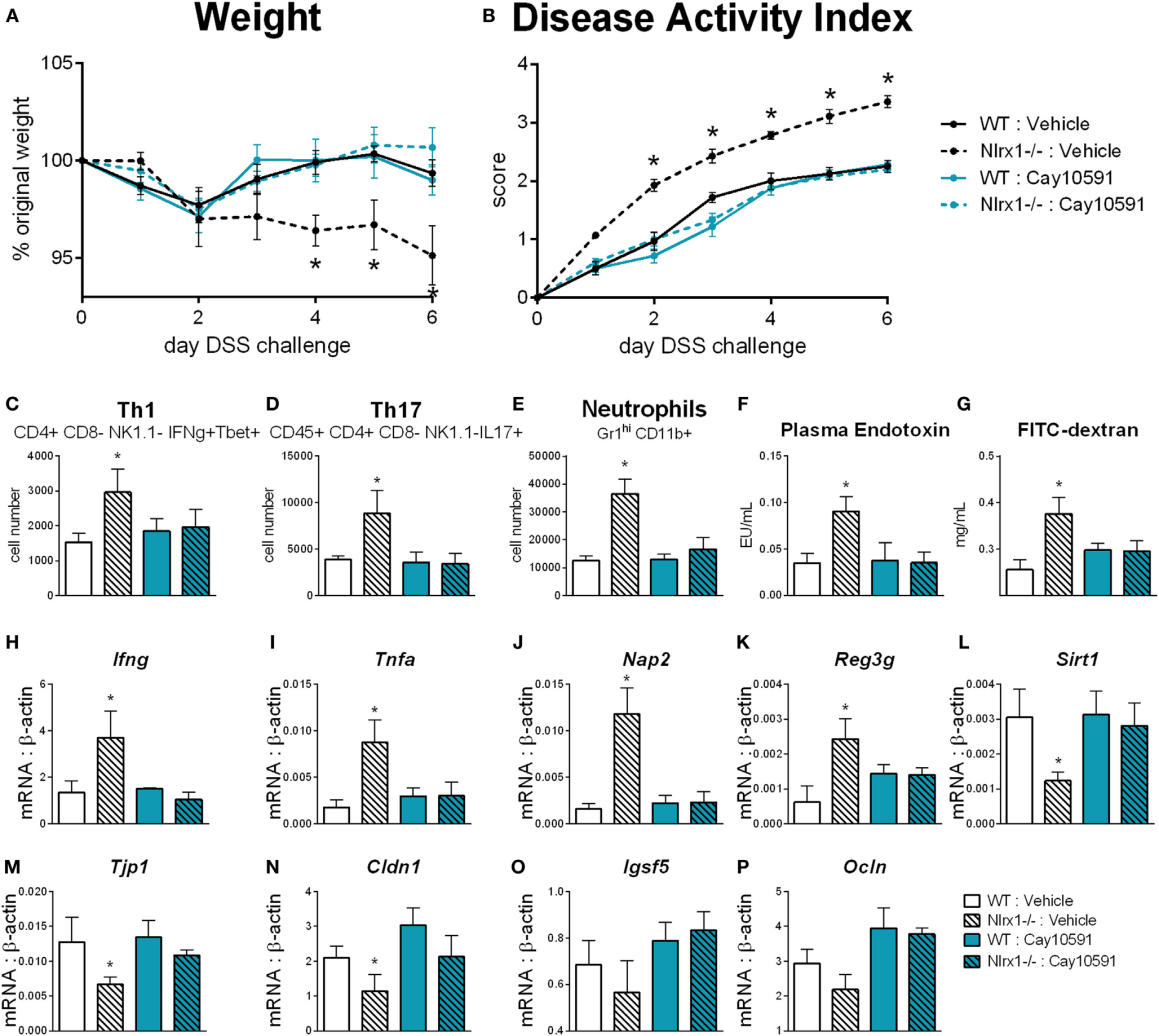
Rescue of SIRT1 activity abrogates differences caused by NLRX1 deficiency. WT and Nlrx1*^−/−^* mice were administered the SIRT1 activator, Cay10591, on days 1, 3, and 6 of DSS challenge by intraperitoneal injection. Weight **(A)** and disease activity index **(B)** throughout course of DSS challenge. Cell number of Th1 **(C)**, Th17 **(D)**, and neutrophils **(E)** within the colonic lamina propria on day 7 of DSS challenge. Plasma endotoxin **(F)** and FITC-dextran **(G)** concentration within heparinized plasma collected 4 h post-FITC-dextran administration by oral gavage. RNA expression of Ifng **(H)**, Tnfa **(I)**, Nap2 **(J)**, Reg3g **(K)**, Sirt1 **(L)**, Tjp1 **(M)**, Cldn1 **(N)**, Igsf5 **(O)**, Ocln **(P)** within whole colon normalized to beta-actin. Asterisks (*) mark significance (*p* ≤ 0.05) in comparison between genotypes (*n* = 9).

We hypothesized that NLRX1 activates SIRT1 activity to promote epithelial cell homeostasis in terms of inflammatory mediator production, maintenance of barrier function and metabolism based on the findings of Figure 5, and the expression changes in the sirtuin family (Figure S6 in Supplementary Material). SIRT1 is a cytosolic and nuclear deacetylase with effects in autophagy, energy, DNA repair, and immunoregulatory pathways. Therefore, we examined the effect of a SIRT1 activator (Cay10591) on WT and *Nlrx1^−/−^* in a DSS model of IBD. Cay10591 effectively rescued SIRT1 expression resulting from the loss of NLRX1 (Figure 8L). The administration of Cay10591 abrogated differences in weight loss and disease activity throughout the course of DSS challenge (Figures 8A,B). On day 7 of DSS challenge, Cay10591 decreased number of Th1, Th17, and neutrophil cell types in the colonic lamina propria (Figures 8C–E) as well as the expression of inflammatory markers *Ifng* and *Tnfa* in whole colon (Figures 8H,I). Cay10591 also diminished differences in representative chemokine and antimicrobial peptide markers (Figures 8J,K), Nap2, and Reg3g respectively, which were previously identified by RNA-seq (Figure 1). Nlrx1*^−/−^* resulted in significantly decreased day 7 colonic expression of tight junction protein 1 (Figure 8M) and claudin 1 (Figure 8N) in addition to suppressed expression of junctional adhesion molecule 4 (Figure 8O) and occludin (Figure 8P). In line with decreased tight junction expression, Nlrx1*^−/−^* mice had higher measures of intestinal permeability (Figures 8F,G), including plasma endotoxin and FITC-dextran, which was prevented by Cay10591 treatment. Blood was collected by cardiac puncture at necropsy 4 h post oral administration FITC-dextran. The abrogation of increased inflammation and disease severity with Cay10591 suggests that NLRX1 might aid in the activation of SIRT1 to control epithelial barrier homeostasis and prevention of intestinal inflammation in response to altered permeability during IBD.

## Discussion

The pathogenesis of IBD encompasses genetic predisposition, immune dysregulation, environmental factors, and gut microbial dysbiosis, although the immunometabolic mechanisms at the interface of these complex multiscale interactions remain to be elucidated. The loss of NLRX1 in IEC is associated with increased disease activity and more severe colonic inflammatory lesions. Transcriptomics, gut microbiome, and host response systems-wide data in NLRX1-expressing and -deficient mice suggest a central role of NLRX1 as an intrinsic sensor of microbial, dietary factors, and metabolic components that might help orchestrate mutual communication between the host responses the gut microbiome.

Decreased colonic NLRX1 levels during the peak-phase followed by rapid recovery of NLRX1 mRNA and protein expression in the late-stage of disease has been acknowledged only recently in the gastric mucosa of mice infected with *Helicobacter pylori*, suggesting that NLRX1 and inflammatory responses are mutually negative regulators (30). NLRX1 negatively regulates NF-κB signaling, thus, it is reasonable to postulate that inflammation-induced suppression of NLRX1 could enhance effector responses and delay tissue repair during IBD (8, 31). Furthermore, our RNA-seq analyses of colonic samples from mice with DSS colitis demonstrate that the loss of *Nlrx1* promotes increased proliferation *via* growth-associated transcriptional activities, apoptotic resistance, and decreased control of the cell cycle suggesting the potential for detrimental post-inflammation effects such as the development of cancer through a loss of growth suppression. Indeed, the findings of increased inflammation in mice with NLRX1-deficient epithelial cells are in line with two reports of the role of NLRX1 in cancer (26, 32). These two publications suggest that NLRX1 functions as a tumor suppressor by decreasing cancer-associated inflammation (IL-6 and TNFα) through effects on NF-κB signaling. Tattoli et al. also depict an increase in epithelial cell proliferation with the loss of NLRX1. In addition to validating the effect of NLRX1 on inflammation and epithelial cell proliferation in a separate model of disease, we elucidate epithelial cell intrinsic pathways that contribute to worsened disease severity and may also help to further define the linkage between NLRX1 and NF-κB signaling or NLRX1 and cancer.

Previously, we have determined that NLRX1 has intrinsic effects on the control and differentiation of CD4+ T cells (11). In IBD models with T cell-initiated disease (adoptive transfer, *C. rodentium*), NLRX1 deficiency results in more severe disease with immunometabolic mechanisms. Within the present manuscript, we focus on an IEC-initiated model of colitis. Critically, both *Nlrx1^−/−^* and *Nlrx1fl/fl*; VillinCre+ respond similarly while the epithelium is being actively damaged. The increased inflammatory cell recruitment, shown through the increased chemokine production, and alterations in barrier function caused by the loss of NLRX1 in IEC create a highly inflammatory mucosal environment that the presence of NLRX1 only in immune cells cannot overcome. However, the two genotypes diverge after DSS administration stops suggesting that NLRX1 deficiency is needed in immune cells to potentiate the impaired recovery of *Nlrx1^−/−^* mice. With independent effects in epithelial and immune cells, NLRX1 may serve as a potent therapeutic target for Crohn’s disease (CD) and ulcerative colitis (UC) due to synergisms of NLRX1 signaling in epithelial and immune cells.

*Nlrx1^−/−^* mice mount a robust antimicrobial response compared to Nlrx1-expressing WT littermate mice. Reg3g, an epithelial-derived antimicrobial peptide with activity against Gram-positive bacteria, is highly upregulated in *Nlrx1^−/−^* challenged with DSS and *WT_Nlrx1_^−/−^* mice (33). In addition to S100A8, Reg3g is highlighted within Figure 1 as a marker of an increased antimicrobial response. While low expression of Reg3g facilitates control of the microbiome in healthy states, elevated expression is highly linked to the presence of inflammation in response to bacterial stimuli or epithelial damage (34). Potentially greater stimulation of TLRs and PAMPs (35) in the absence of NLRX1 and by the NLRX1*^−/−^* associated microbiome may induce Reg3g expression, making Reg3g a viable marker of NLRX1 activity *in vivo*. The complete RNA-seq dataset supports a broader increase in the production of antimicrobial peptides. While potentially effective in reducing harmful and invading bacteria, the increased antimicrobial response can also reduce the presence of beneficial commensal bacterial strains. This loss of commensals can further reduce regulatory responses exacerbating the inflammatory environment. Notably, the differential patterns in *Nlrx1^−/−^* compared to *Nlrx1*-expressing mice in prominent antimicrobial peptides, S100A8/A9, Reg3g, and Defb1, follow the patterns observed in patients experiencing active IBD suggesting that a suppression of NLRX1 might be implicated in mediating disease and pathology in CD and UC patients (36, 37).

NLRX1 plays a critical role in controlling lactate metabolism and effector responses in CD4+ T cells of mice with IBD (11). Consistent with our previous findings, our current study suggests that NLRX1 is a regulator of overall cellular metabolism in addition to its role in immunoregulation. With an increased demand and consumption of nutrients and metabolites, *Nlrx1^−/−^* cells may shape the gut microbiome through controlling availability of metabolic substrates. Paramount among these factors are amino acids, specifically, the conditionally essential amino acid glutamine. The concept that immune and metabolic pathways are intertwined and have important codependencies has a clear relevance to the understanding of disease and therapeutic development, particularly in IBD (38, 39). Differences in the use of metabolic resources for cellular proliferation at the IEC level can result in altered barrier integrity and chemokine secretion profiles (40). The fate of metabolic intermediates is crucial to the balance of energy producing and biosynthetic pathways, such as those related to the production of large quantities of cytokines and chemokines (41). Glutamate and glutamine are critical focal points of this balance, capable of being converted to α-ketoglutarate for the augmentation of the tricarboxylic acid (TCA) cycle or to other amino acids for the biosynthesis of protein and nucleic acids. Based on the previous mechanistic linkage of NLRX1 to the regulation of the TCA cycle (11), it is reasonable to suggest that NLRX1 may help to couple glutamine metabolism to the TCA cycle.

The variation in amino acid availability through diet, host metabolism, and competitive microbial species is an important factor in shaping the composition of the intestinal microbiome (42). The metabolism and synthesis of amino acids is an important metagenomic signature of the distal gut microbiome (43). While our data focuses on l-glutamine, the host–microbiome interactions on amino acids extend beyond this scope. For example, asparagine, arginine, and tryptophan have been identified to be crucial in the control of notable pathogens *Mycobacterium tuberculosis* (44), *Staphylococcus aureus* (45), and enteroaggregative *Escherichia coli* (46), respectively. However, in particular, l-glutamine dietary supplementation decreases *Dorea* and *Veillonella* while decreasing the overall *Firmicutes* to *Bacteroidete*s ratio in humans (29). Beyond strictly microbial effects, glutamine supplementation has also been linked to decreased production of secretory immunoglobulin A (47) and suppressed intestinal inflammation (48). As such, we identify through both oral supplementation with glutamine and inhibition of its use by the host effectively restore the *Nlrx1^−/−^*-associated gut microbiome and resolve differences in disease severity and immune response during DSS colitis. Thus, the immunometabolic effects of NLRX1 in glutamine metabolism control its effects in intestinal inflammation.

Many of these microbial changes observed in NLRX1*^−/−^* mice have also been linked to the gut microbiomes associated with CD, primary sclerosing cholangitis, and other digestive inflammatory disorders, specifically, the downregulation of Bacteroidetes and the expansions of *Veillonella* and *Porphyromonas* (49–51). That *Nlrx1^−/−^* mice harbor bacteria with a higher connection to inflammatory mechanisms may contribute to an increased disease severity in IBD. When the Nlrx1*^−/−^*-associated colonic microbiome is transferred to WT mice, disease severity worsens as evidenced by weight loss, colonic lesions, and increased prevalence of inflammatory cell types and disease biomarkers. It is plausible to speculate that with the altered gut microbiome reduces the availability of microbial-derived NLRX1 ligands locally in the gut mucosa, accelerating downregulation of NLRX1 and exacerbating effector responses. Notable depleted taxa include *Faecalibacteria* and *Akkermansia*. *Faecalibacteria* is an important butyrate producer that is in lower abundance within UC patients with secreted product profiles capable of decreasing NF-κB activity (52, 53). *Akkermansia*, aside from its well-characterized mucin-degrading ability, is also associated with anti-inflammatory effects in IBD patients with inductions of lipid metabolism, peroxisome proliferator-activated receptor signaling, and modulation of antigen presentation (54–56). Further exploration of the mechanistic and translational linkages between these two bacteria and NLRX1 may reveal a new molecular biosignature that allows communication between the gut microbiome and host immunometabolism through NLRX1.

Coinciding with the elucidation of these downstream effects, we identify that NLRX1 deficiency alters NAD+ concentrations within epithelial cells. Numerous important signaling molecules are dependent on NAD+ as a co-factor in their mechanisms of action, such as PARP1, which is involved within DNA damage repair (57) and could be an additional linkage between NLRX1 and cancer. Sirtuins are a main family of these molecules (58, 59). SIRT1, in particular, was identified to be influenced by the absence of NLRX1 in Figures 5 and 8 of this manuscript. While unexplored currently, NLRX1 may also impact other sirtuins, such as the mitochrondrial sirtuin, SIRT3, which has been linked to ROS production. Importantly, the majority of effects of SIRT1 deficiency overlap with those of NLRX1 in experimental models of IBD (59) and similarly influence STAT3 and NF-κB pathways (32, 60). SIRT1 has been connected to metabolic effects including glutamine dehydrogenase (61) as well as the maintenance of tight junction expression (62). The dysregulation of SIRT1 rescued by the SIRT1 activator Cay10591 (62) is linked to altered permeability of the GI tract. Combined with the increased glutamine utilization, the gut microbiome of *Nlrx1^−/−^* mice can shift and have increased likelihood for translocation across the epithelial barrier. This translocation can lead to the increased production of TNFα, chemokines, and antimicrobial peptides at the gut mucosa. Due to the lack of NLRX1-mediated regulation, this inflammation can persist with impaired resolution and an inability to restore intestinal homeostasis.

In summary, epithelial NLRX1 ameliorates intestinal inflammation during experimental IBD and maintains a healthy gut microbiome *via* sensing of microbial and nutritionally derived lipid signals. The activation of NLRX1 results in downstream modulation of IEC-derived chemokines and inflammatory cytokines driving the recruitment and stimulation of leukocytes early in disease. Previously, we had demonstrated the importance of NLRX1 in the interface of immunity and carbohydrate metabolism in CD4+ T cells, this study broadens our understanding of the immunometabolic roles of NLRX1 to include the control of amino acid metabolism leading to the changes in the gut microbiome, IEC proliferation, and effector responses. Further mechanistic understanding of the immunometabolic mechanisms of NLRX1 in IBD is required to validate its potential as a new therapeutic target for UC and CD.

## Materials and Methods

### Experimental Model of Colitis

C57BL/6 wild-type (WT), *Nlrx1^−/−^, Nlrx1fl/fl*, and *Nlrx1fl*/*fl*; VillinCre+ mice ranging from 7 to 10 weeks of age were administered DSS in drinking water for 7 days. *Nlrx1*-expressing designation is used when WT and *Nlrx1fl*/*fl* mice were used. No significant differences have been observed between these genotypes as both have unaltered expression of NLRX1. Control mice received tap water. On days 3 and 7, mice were euthanized *via* carbon dioxide narcosis with secondary cervical dislocation. All mice were weighed and scored daily. Clinical scores were based on physical appearance (0–3), fecal consistency (0–3), presence of rectal bleeding (0–4), and weight loss (0–3) and assigned a compounded score for overall disease activity (0–4). For l-glutamine supplementation, mice were gavaged daily with 375 mg/kg l-glutamine in PBS, starting from 7 days prior to DSS challenge and continuing through disease time course. Control mice were gavaged with 375 mg/kg alanine in PBS during the same time period. For 6-diazo-5-oxo-l-norleucine (DON) treatment, mice were injected intraperitoneally with 1.3 mg/kg DON in PBS every 3 days, starting 6 days prior to DSS challenge and continuing through the disease time course. All experimental procedures and methods were reviewed and approved by the Virginia Tech (protocol #: 14-013) and Biotherapeutics, Inc. (protocol #: 16-008) IACUC.

### Antibiotic Reduction of Native Microbiome

Eight-week-old mice were administered an antibiotic cocktail in drinking water containing colistin (4.2 mg/kg), gentamycin (3.5 mg/kg), metronidazole (21.5 mg/kg), and vancomycin (4.5 mg/kg) based on estimated water consumption rates for 3 days (63). Mice were then returned to standard drinking water. One day following the conclusion of the antibiotic water, mice were given an intraperitoneal injection of clindamycin (32 mg/kg). The next day, mice were placed within cages containing mice of the opposite genotype that had not received antibiotics in a 1:1 ratio. DSS challenge was started 2 weeks after initiation of cross-housing.

### Histopathology

Colonic samples were fixed in 10% buffered formalin, embedded in paraffin, processed routinely, and sectioned at 5 µm. Sections were stained with hematoxylin and eosin and then examined and graded using an Olympus microscope and ImagePro software. Sections were graded for leukocytic infiltration (0–4), epithelial erosion (0–4), and mucosal thickness (0–4).

### Gene Expression

Total RNA was isolated from mouse colonic contents using a Qiagen RNA isolation mini kit. Complementary DNA (cDNA) was generated from each sample using the iScript cDNA synthesis kit. Standards were produced through a polymerase chain reaction on the cDNA with Taq DNA polymerase from Invitrogen. The amplicon was purified using the Mini-Elute PCR purification kit from Qiagen. Expression levels were obtained through quantitative real-time PCR on a Bio-Rad CFX 96 Thermal Cycler using the Bio-Rad SYBR Green Supermix. For analysis, the starting amount of antimicrobial peptide cDNA was compared to that of beta-actin, as a control. Primer sequences are provided in supplemental information. Primers for bacterial 16S validation and targeted analysis were as follows: *Veillonella* (F: GACGGCCTTCGGGTTGTAAAG, R: TTCCGGTACCGTCAATCCTTCT), *Porphyromonas* (F: GGAAGAGAAGACCGTAGCACAAGGA, R: GAGTAGGCGAAACGTCCATCAGGTC), *Faecalibacterium* (F: CCATGAATTGCCTTCAAAACTGTT, R: GAGCCTCAGCGTCAGTTGGT), *Akkermansia* (F: CAGCACGTGAAGGTGGGGAC, R: CCTTGCGGTTGGCTTCAGAT), total bacteria (F: GCCAGCAGCCGCGGTAA, R: GACTACCAGGGTATCTAAT).

### RNA Sequencing

RNA was submitted for whole transcriptome gene expression assessment using Illumina Hiseq (Virginia Bioinformatics Institute Core Lab Facilities). Fastq files containing 100 bp-long pair-end reads were assessed and poor quality reads (>40% of bases with PHRED score <10; percentage of N greater than 5%; and polyA reads) were filtered out. Remaining high quality reads were mapped to RefSeq (mm10 from http://genome.ucsc.edu/) using Bowtie (version: 1.0.0) with parameters set to “-l 25-I 1-X 1000-a-m 200.” Gene expression levels were calculated using an expectation-maximum algorithm, RSEM. FPKM (fragments per kilobase per million sequenced reads) values were used as expression level measurements. The resultant dataset has been submitted to the NCBI GEO database (accession number GSE68419).

### 16S rRNA Sequencing

A total of 11,362,951 of reads remained after quality filtering and adapter trimming, averaging 757,530 ± 121,290 per sample over 15 samples. Of the paired reads generated, the read-1 sequences were found to completely span the amplicon, were of high quality, and were used for the analysis to avoid losses incurred when overlapping the pairs. The set of all trimmed reads was subjected to dereplication, which found 321,566 unique sequences. The sequences with at least three observations were subjected to chimera filtering and operational taxonomic unit (OTU) clustering using cluster_otus option of the usearch program using the 97% similarity threshold. This yielded 3,530 clusters or OTUs. The centroids of 1,274 larger OTUs with at least 10 reads were used as a reference database, and all reads for each sample were searched against this to match reads to OTUs at a minimal similarity of 97%. A total of 93.5% of reads were members of OTUs. Sequences were searched against the GreenGenes reference ssuRNA data set (release 13.8, clustered at 97% similarity). For each OTU, the most specific taxon, which was common to at least 80% of the sampled reads was chosen as the best taxonomic descriptor.

### Intestinal Organoid Culture

Colon and small intestine were collected from healthy WT and NLRX1*^−/−^* mice. Samples were cut into 1 mm sections and shaken within Gentle Cell Dissociation reagent for 30 min. Sections were washed and fractions were generated by gentle pipetting. Crypt suspensions were centrifuged and adjusted to 1,000 crypts/mL by microscopic evaluation. Aliquots of 1,000 crypts were resuspended in 300 μL of a 50:50 mixture of Matrigel and supplemented MEM. 50 μL of mixture was plated in a 12-well plate and allowed to solidify for 10 min. Additional complete MEM was added to each well to cover the Matrigel dome. Media was exchanged every 3 days. Samples were stimulated with LPS (100 ng/mL) and respective treatments on day 7 post-plating. Proliferation, metabolic and expression assays were conducted after 24 h of LPS stimulation.

### Statistics

For the analysis of the RNA-seq dataset, a two-way (genotype and treatment) was performed in R. Normal quantile transformation was used to normalize the FPKM to fit the normality assumption of ANOVA (tested with Kolmogorov–Smirnov test). FDR and Bonferroni adjustments were used to identify differentially expressed genes. For analysis of standard data, a one-way (genotype) ANOVA was performed to determine significance in the data using a SAS (SAS Institute) general linear model procedure. Differences of *p* ≤ 0.05 were considered significant. The number of samples for each group varied between five and eight. Data are displayed as mean values with error bars representing SEM and asterisks to indicate statistical significance.

## Ethics Statement

All experimental procedures were approved by the Institutional Animal Care and Use Committee (IACUC) of Virginia Tech and Biotherapeutics, Inc., and met or exceeded requirements of the National Institutes of Health and the Animal Welfare Act. The IACUC approval IDs for the study were 14-013-VBI and 16-008.

## Author Contributions

AL, NT-J, and VZ-R contributed to the generation of biological data. AL and VA contributed to the processing and analysis of sequencing data. AL, RH, and JB-R conceived study designs and wrote the manuscript.

## Conflict of Interest Statement

AL, RH, VZ-R, and JB-R are employees of Landos Biopharma, Inc. All other authors declare that the research was conducted in the absence of any commercial or financial relationships that could be construed as a potential conflict of interest.
