# Supplementary material for: NLRX1 Modulates Immunometabolic Mechanisms Controlling the Host–Gut Microbiota Interactions during Inflammatory Bowel Disease
Source: Front Immunol. 2018 Feb 26;9:363. doi: 10.3389/fimmu.2018.00363 (PMC5834749; doi:10.3389/fimmu.2018.00363)
Supplement: Supplementary file 1 [file image_1.PDF]

SUPPLEMENTAL INFORMATION

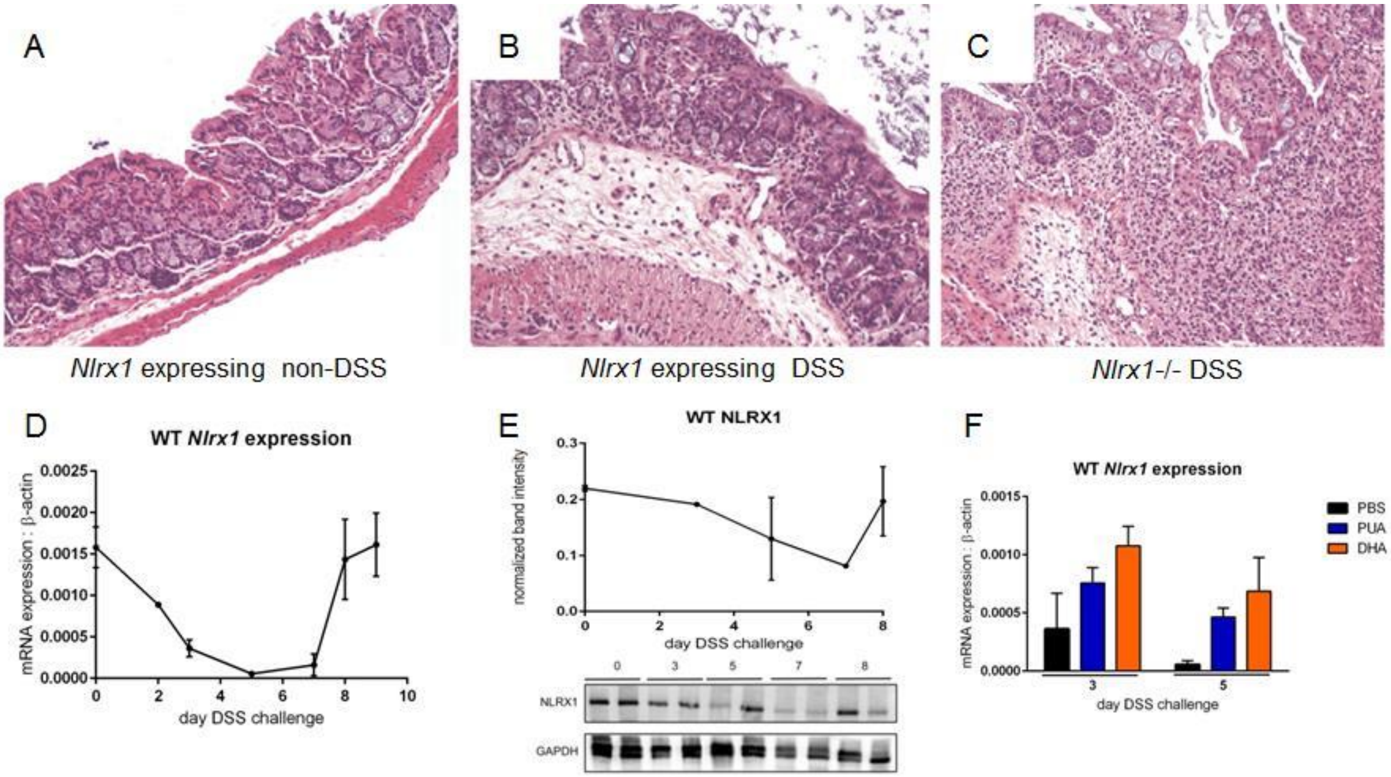

Figure S1. Histological implications of NLRX1 deficiency and temporal patterns of NLRX1 expression. H&E stained representative photomicrographs of colons from Nlrx1 expressing non-DSS (A), Nlrx1 expressing DSS (B), and Nlrx1-/- DSS (C). mRNA (D) and protein (E) level expression of NLRX1 in colon during DSS challenge. mRNA expression of Nlrx1 (F) after treatment with punicic acid (PUA) and docosahexaenoic acid (DHA).

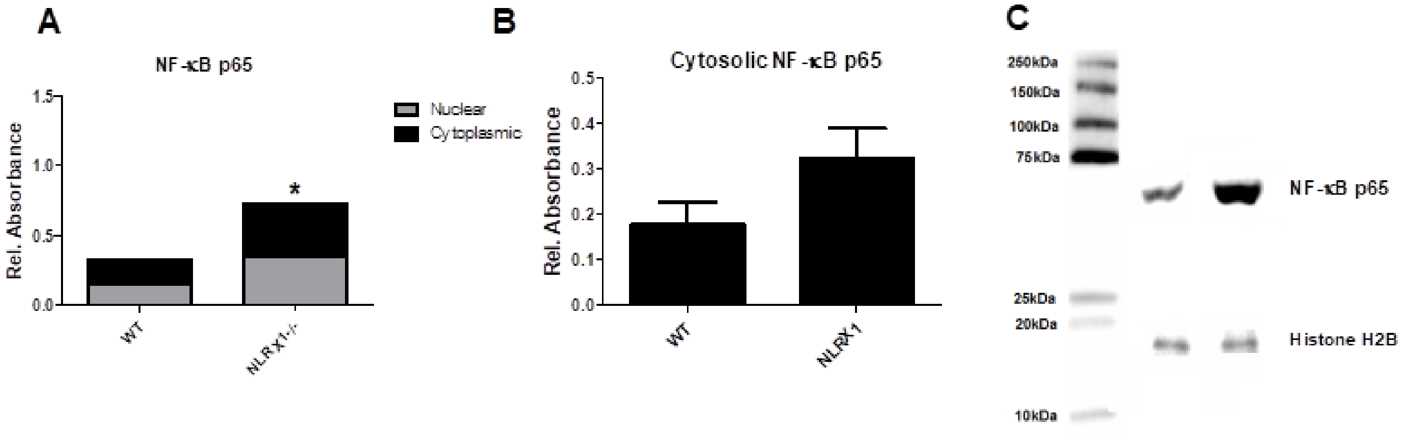

Figure S2. NF-kB in WT and *Nlrx1-/-* in vivo. *Nlrx1-/-* mice displayed significantly increased concentrations of intranuclear (i.e. activated) NF-κB ($P \leq 0.05$) as well as increased overall cytosolic NF-κB ($P \leq 0.03$) within whole colon homogenates via ELISA (A, B, n=10). Nuclear translocation and activation of NF-κB change was further confirmed upon Western blot of nuclear extracts where increased intranuclear NF-κB (3C) was seen. Histone H2B was used as a loading control.

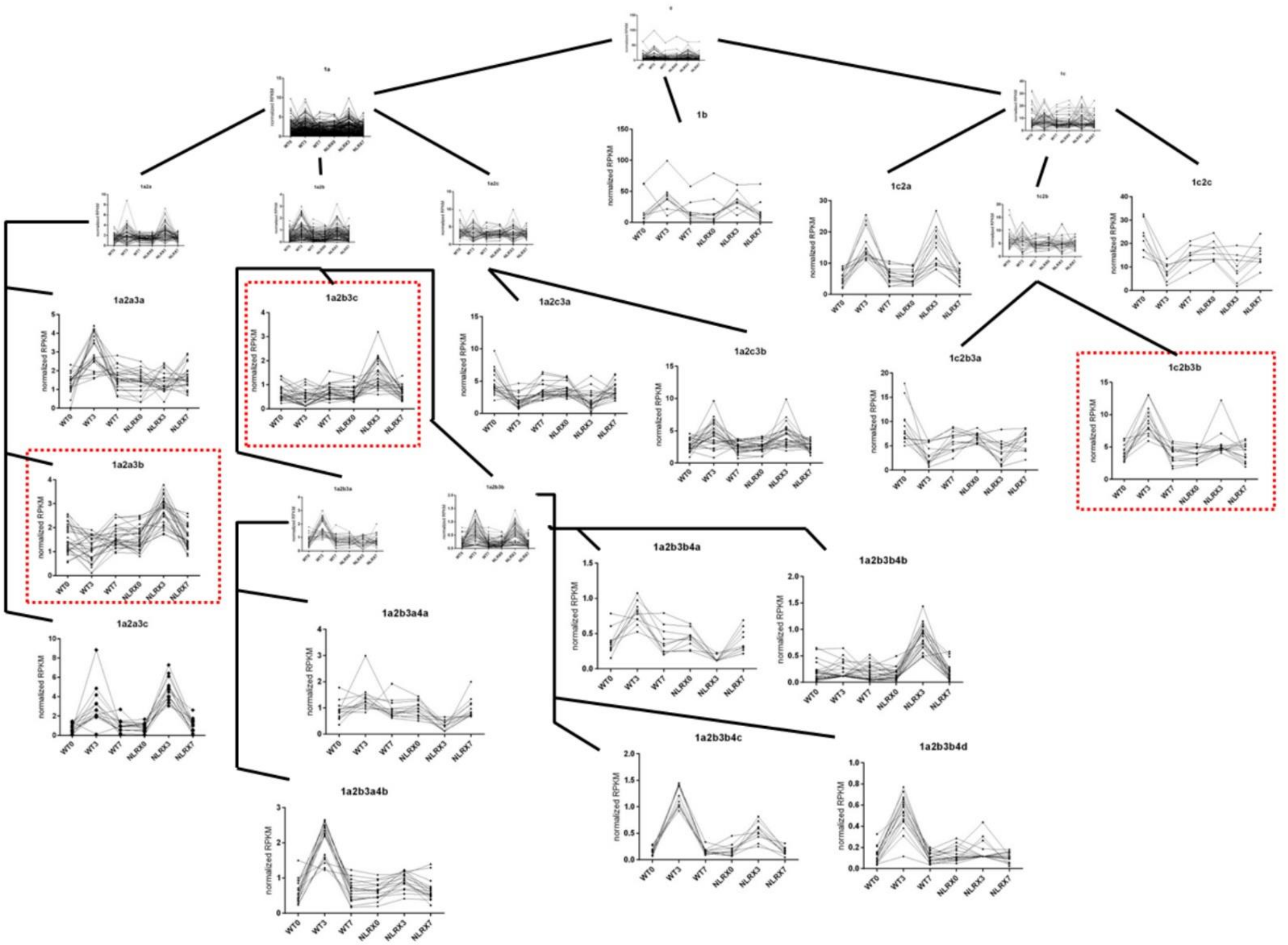

Figure S3. Hierarchical clustering of genes significant (p<0.05) by genotype-time interaction. From RNA sequencing analysis for day 0, 3 and 7 of DSS challenge in *Nlrx1* expressing and *Nlrx1*-/- mice, 301 significant genes were identified. Using the cmeans function for fuzzy clustering in the e1071 R package, genes were grouped into clusters by dynamic pattern through a multi-step process. Final clusters were analyzed via Gene Ontology for shared functions. Cluster boxed in red possessed fold enrichments greater than 4 for functions within the cell life cycle. Cluster 1a2b3c, showing a flat trend within *Nlrx1* expressing and an increasing trend in *Nlrx1*-/-, is enriched for proliferative and mitotic functions via genes *Adm*, *Skp2*, *Ska3*, *Paf1*, *Nup37*, *Nrf1*, and *Pdgfb*. Cluster 1a2a3b, showing a decreasing trend within *Nlrx1* expressing and an increasing trend in *Nlrx1*-/-, is enriched for differentiation and cell cycle functions via genes *Carm1*, *Nupl1, Igf2bp2*, *Bcl2l2*, and *Klhl22*. Cluster 1c2b3b, showing an increasing trend within *Nlrx1* expressing and a flat trend in *Nlrx1*-/-, is enriched for cell death functions via genes *Pdcd5*, *Camk1d*, *Mad2l1*, *Cflar*, and *Cd34*.

Effect of NLRX1 on the control of cell life cycle and proliferation. After hierarchical clustering three of the fifteen clusters were enriched in cell lifecycle-associated functions. Two of these groupings displayed an upregulation of genes within *Nlrx1*-/- compared to *Nlrx1* expressing, while the third displayed a downregulation. Combined, the two *Nlrx1*-/- upregulated clusters identified an increased prevalence of proliferation and cell division through growth factors (*Igf2bp2*, *Pdgfb*), transcription factors promoting NF-κB and Wnt signaling (*Carm1*, *Paf1*) and factors responsible for progressing the cell cycle (*Klhl22*, *Skp2*, *Ska3*). In addition, the two clusters also contained oncogenes and apoptosis resistance factors (*Skp2*, *Ska3*, *Bcl2l2*). In contrast, the *Nlrx1*-/- downregulated clusters contained inhibitors of cell cycle progression (*Mad2l1*) and apoptosis promoting factors (*Pdcd5*, *Cflar*). Therefore, the loss of *Nlrx1* may have detrimental post-inflammation effects such as the development of cancer through a loss of growth suppression or granuloma formation through uncontrolled proliferation.

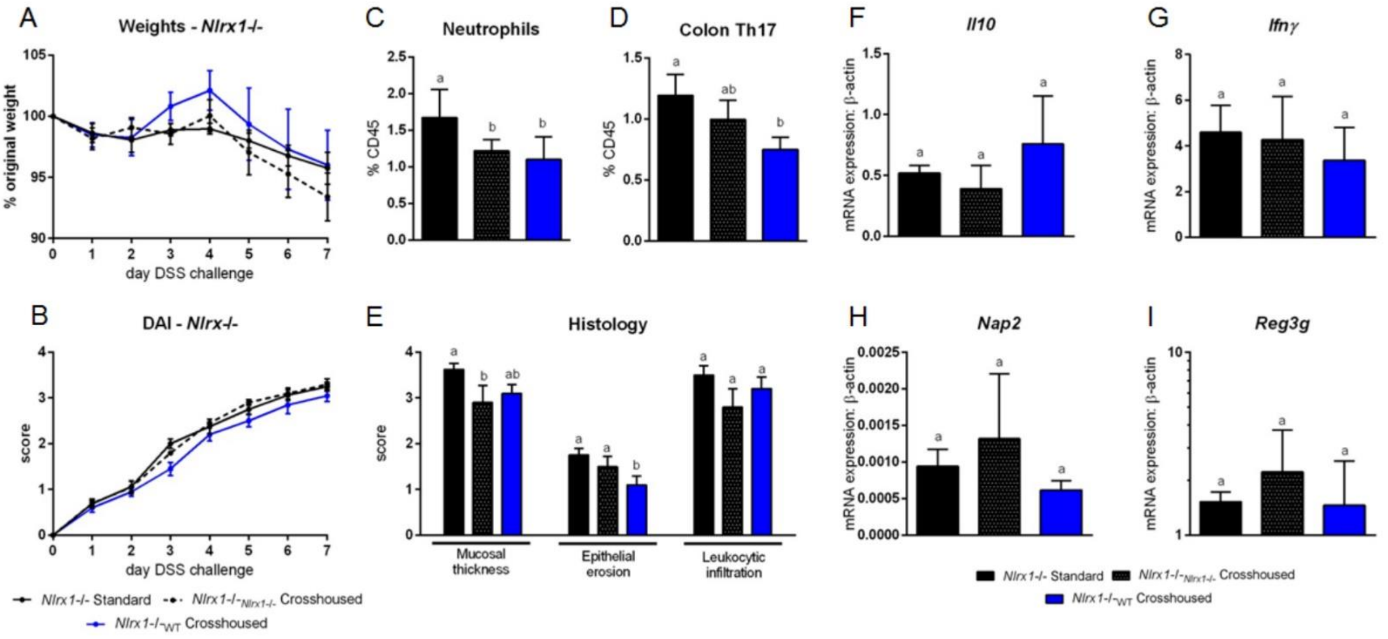

Figure S4. WT microbiome transfer fails to ameliorate severity in *Nlrx1-/-* mice. Weight change (A) and disease activity index (B) of Nlrx1-/- mice following cross-cohousing. Colonic neutrophil (C) and Th17 (D) responses on day seven of DSS challenge. Colonic histological scores (E) for mucosal thickness, epithelial erosion and leukocytic infiltration criteria on day seven of DSS challenge. Expression of Il10 (F), Ifnγ (G), Nap2 (H), and Reg3g (I) mRNA by qRT-PCR assay of colon samples on day seven of DSS challenge. Asterisks (*) mark significance ($p \leq 0.05$) in comparison between treatments within genotypes, letters (a,b,c) mark statistical grouping by ANOVA ($n = 6$).

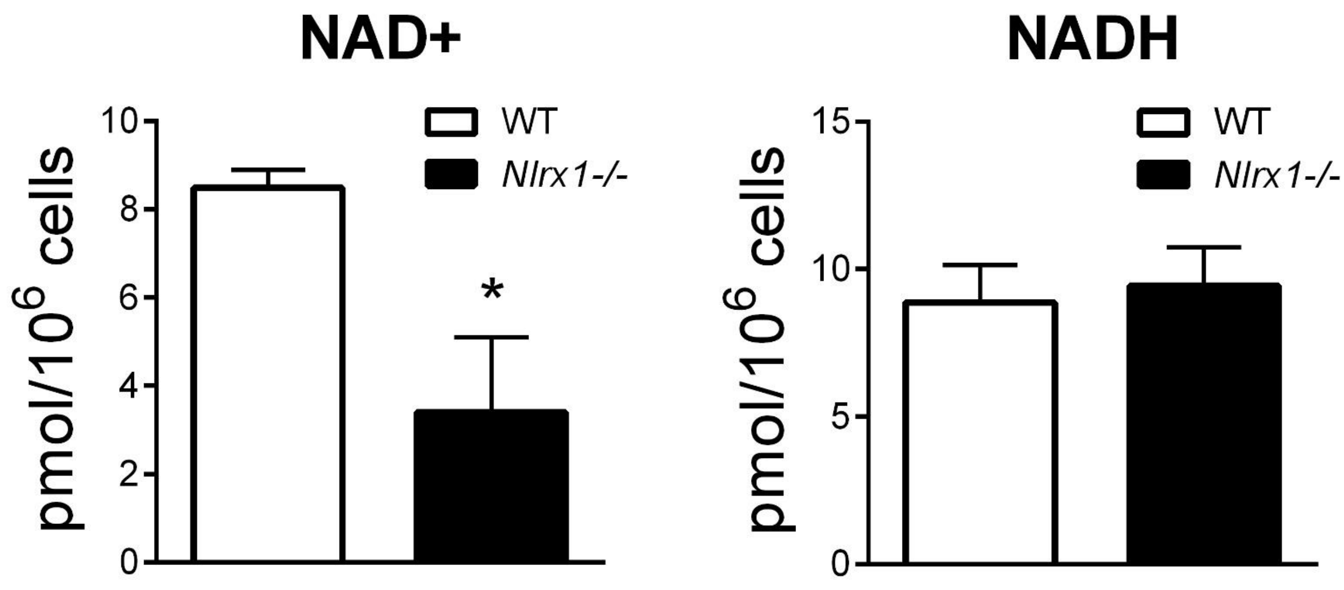

Figure S5. NAD+ concentration *in vivo*. NAD+ and NADH concentrations normalized to cell number in isolated colonic epithelial cells on day 7 of DSS challenge. Asterisks (*) mark significance ($p \leq 0.05$) in comparison between genotypes (n = 9).

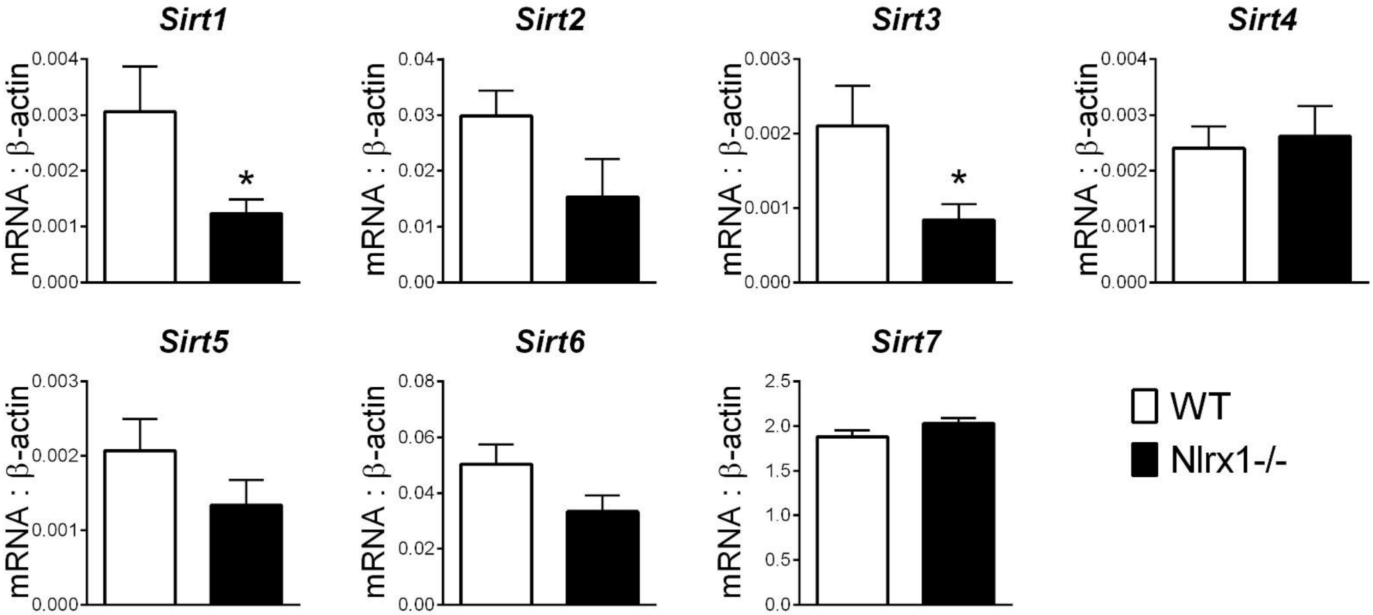

Figure S6. Sirtuin expression in vivo. mRNA was isolated from whole colon on d 7 of DSS challenge. Expression of SIRT1-7 was measured by qRT-PCR and normalized to beta-actin expression. Asterisks (*) mark significance ($p \leq 0.05$) in comparison between genotypes ($n = 5$).

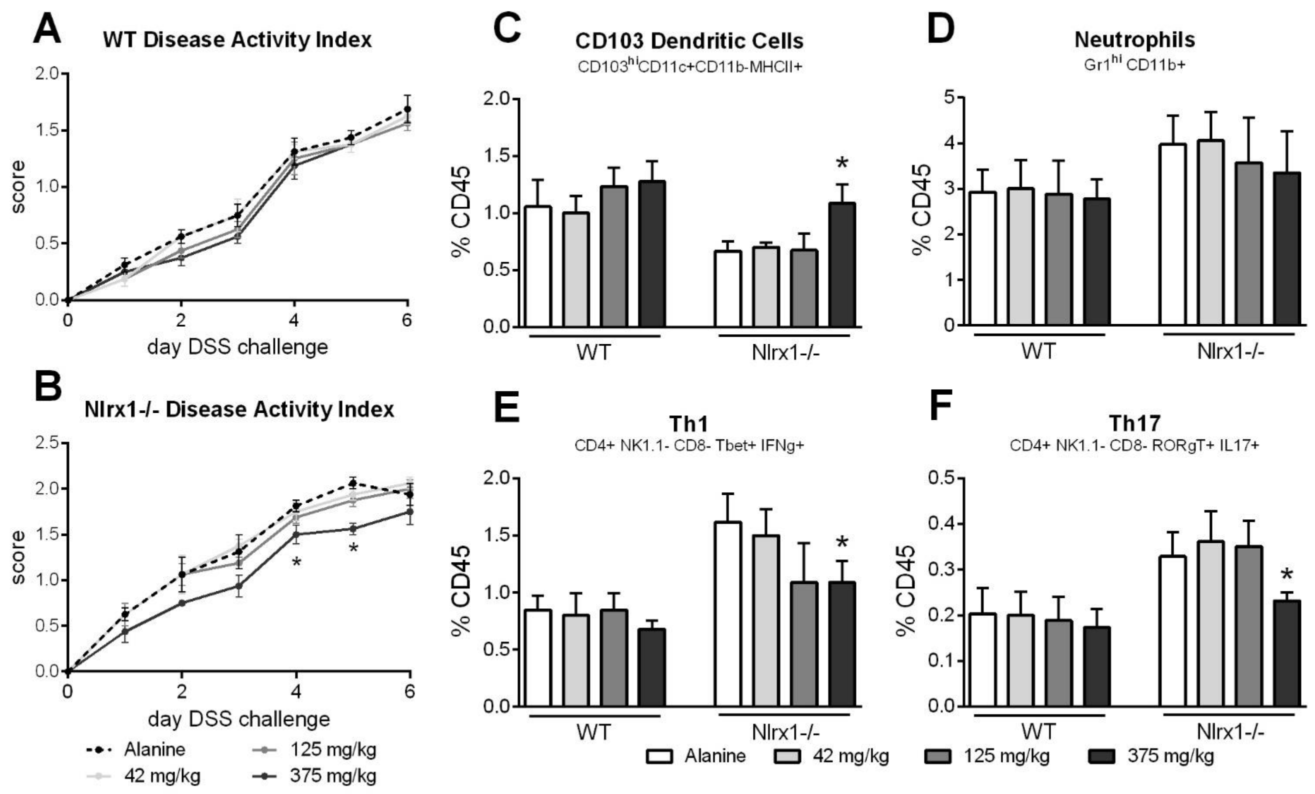

Figure S7. Immunological dose titration of glutamine. Glutamine was delivered orally at three doses (42, 125, 375 mg/kg) daily over 7 d DSS challenge. Oral alanine was used as a negative control. Disease activity was scored daily for wild type (A) and Nlrx1-/- (B) mice. At d 7, colons were digested and immunophenotyped for CD103+ dendritic cells (C), neutrophils (D), Th1 (E) and Th17 (F) cells by flow cytometry. Asterisks (*) mark significance ($p \leq 0.05$) in comparison between genotypes (n = 8).
